# Preserved performance monitoring and error detection in left hemisphere stroke

**DOI:** 10.1016/j.nicl.2020.102307

**Published:** 2020-06-10

**Authors:** Eva Niessen, Jana M. Ant, Stefan Bode, Jochen Saliger, Hans Karbe, Gereon R. Fink, Jutta Stahl, Peter H. Weiss

**Affiliations:** a Cognitive Neuroscience, Institute of Neuroscience and Medicine (INM-3), Research Centre Jülich, Germany; b University of Cologne, Faculty of Medicine and University Hospital Cologne, Department of Neurology, Germany; c Melbourne School of Psychological Sciences, University of Melbourne, Australia; d Department of Individual Differences and Psychological Assessment, University of Cologne, Germany; e Neurological Rehabilitation Centre Godeshöhe, Germany

**Keywords:** Stroke, Cognitive control, EEG, Multivariate pattern analysis, Event-related potentials

## Abstract

Depending on the lesion site, a stroke typically affects various aspects of cognitive control. While executing a task, the performance monitoring system constantly compares an intended action plan with the executed action and thereby registers inaccurate actions in case of any mismatch. When errors occur, the performance monitoring system signals the need for more cognitive control, which is most efficient when the subject notices errors rather than processing them subconsciously. The current study aimed to investigate performance monitoring and error detection in a large sample of patients with left hemisphere (LH) stroke.

In addition to clinical and neuropsychological tests, 24 LH stroke patients and 32 healthy age-matched controls performed a Go/Nogo task with simultaneous electroencephalography (EEG) measurements. This set-up enabled us to compare performance monitoring at the behavioral and the neural level. EEG data were analyzed using event-related potentials [ERPs; e.g., the error-related negativity (Ne/ERN) and error positivity (Pe)] and additionally more sensitive whole-brain multivariate pattern classification analyses (MVPA). We hypothesized that LH stroke patients would show behavioural deficits in error detection when compared to healthy controls, mirrored by differences in neural signals, in particular reflected in the Pe component.

Interestingly, despite clinically relevant cognitive deficits (e.g., aphasia and apraxia) including executive dysfunction (trail making test), we did not observe any behavioral impairments related to performance monitoring and error processing in the current LH stroke patients. Patients also showed similar results for Ne/ERN and Pe components, compared to the control group, and a highly similar prediction of errors from multivariate signals. ERP abnormalities during stimulus processing (i.e., N2 and P3) demonstrated the specificity of these findings in the current LH stroke patients. In contrast to previous studies, by employing a relatively large patient sample, a well-controlled experimental paradigm with a standardized error signaling procedure, and advanced data analysis, we were able to show that performance monitoring (of simple actions) is a preserved cognitive control function in LH stroke patients that might constitute a useful resource in rehabilitative therapies for re-learning impeded functions.

## Highlights

• Our patients suffered from severe cognitive deficits and executive dysfunction.
• On the Go/Nogo task, stroke patients expressed no behavioral impairments.
• Patients showed no neural abnormalities in performance monitoring (Ne/ERN and Pe)
• However, patients had neural abnormalities during stimulus processing (N2 and P3)
• Preserved cognitive control function might be useful tool for rehabilitation.

## 1 Introduction

After suffering a stroke, patients have to strain themselves to regain their stroke-impaired functions through rehabilitation. Aside from impairments due to primary sensorimotor deficits (i.e., paresis, sensory loss), less obvious cognitive deficits often impede rehabilitation. For example, left hemisphere (LH) stroke patients may show inhibitory control deficits (Krämer et al., 2013). The presence of such stroke-related motor-cognitive deficits and their consequences (e.g., lack of inhibition and difficulties with action planning) can profoundly impact on the accuracy of executed movements and the occurrence of action errors. Moreover, putative cognitive impairments, such as reduced awareness of one’s actions, most likely interfere with an effective re-learning of impaired motor and cognitive functions due to LH stroke. From studies with healthy participants, it is well-known that when a participant consciously detects that a behavior was not as intended (i.e., an error occurred), these errors are monitored and behavioral adjustments are implemented to prevent future errors (Ridderinkhof et al., 2004). Consequently, measurable behavioral adjustments, such as slower and more careful responding (e.g., Danielmeier and Ullsperger, 2011), are assumed to reflect successful error processing. These adjustments also indicate increased cognitive control, as demanded by the performance monitoring system after an erroneous action. If cognitive control functions like performance monitoring fail, patients will have difficulties adjusting their behavior, resulting in a worse (rehabilitation) outcome. Hence, it is essential to investigate the processes of performance monitoring and error detection in stroke patients.

Two components of the event-related potential (ERP) allow assessing the neural correlates of performance monitoring and error processing. The error(-related) negativity (Ne/ERN; Falkenstein et al., 1991, Gehring et al., 1993), typically measured at central midline electrodes as a negative signal deflection, peaks within 50 to 100 ms after an erroneous response and is generated by the dorsal part of the anterior cingulate cortex (dACC; Debener et al., 2005, Dehaene et al., 1994, Roger et al., 2010). The Ne/ERN is supposed to reflect the rapid, automatic processing of an incorrect action signaling the demand for increasing cognitive control to other brain regions (for review see Gehring et al., 2012). The second component, the error positivity (Pe), a positive voltage deflection measured at more posterior midline electrodes between 150 and 300 ms, immediately follows the Ne/ERN. It is usually observed when participants detect their own errors (Falkenstein et al., 1991, Falkenstein et al., 1994), which is why this component has been discussed as an indicator of error awareness (Nieuwenhuis et al., 2001) and error evidence accumulation (Steinhauser and Yeung, 2012). In addition to the response-locked ERPs Ne/ERN and Pe, it is important to assess two stimulus-locked components, namely the N2 and P3, when trying to understand how participants process cognitive tasks. While the N2 is often associated with the evaluation and processing of conflicting or difficult stimuli and the respective inhibitory processes (e.g., in the case of nogo stimuli) (Cheng et al., 2019, Nieuwenhuis et al., 2003), the P3(a) has been suggested to be involved in signaling the need for more cognitive control, in case a conflict has been detected earlier (Polich, 2007). Thus, both components represent important cognitive processes whose output (e.g., identifying a nogo stimulus necessitating a high degree of cognitive control) is of high relevance for the performance monitoring system.

Previous studies on performance monitoring and error processing in stroke patients revealed an inconsistent pattern concerning the Ne/ERN and Pe (Stemmer et al., 2004). Supplementary Table 2 presents an overview of the results from previous studies. While some studies reported a similar neural processing of errors for stroke patients and healthy controls (Maier et al., 2015, Ullsperger et al., 2002), most studies found abnormalities for their patient sample (or for one of their patient sub-samples in case of multiple groups) (Gehring and Knight, 2000, Maier et al., 2015, Swick and Turken, 2002, Turken and Swick, 2008, Ullsperger et al., 2002, Ullsperger and von Cramon, 2006, Wessel et al., 2014). These abnormalities were mostly due to elevated correct-related negativity (CRN) leading to a reduced differentiation between correct responses (i.e., CRN) and errors (i.e., Ne/ERN). Two of these studies additionally assessed the subjective detection of errors, and both concluded that, despite other impairments, patients showed no deficits in error detection (Maier et al., 2015, Stemmer et al., 2004). However, caution is warranted when considering these results. Sample sizes in previous studies were generally rather small (maximum of 9 subjects per patient group) since recruitment focused on patients with specific lesion location (e.g., lesions restricted to the ACC). Furthermore, the influence of cognitive deficits (as assessed with neuropsychological tests) on markers of performance monitoring was not investigated. In addition to assessing performance monitoring, the applied tasks in some studies put high demands on other cognitive functions (e.g., working memory) (Fellows, 2017, McCabe et al., 2010). Finally, concerning the assessment of error detection, standardized procedures were missing [e.g., the experimenters manually took notes of the patients’ behavior/ utterances (Maier et al., 2015, Stemmer et al., 2004)]. The current study therefore aimed at examining error detection in left hemisphere stroke patients by using a Go/Nogo task with a standardized but simple procedure for error signaling as previously adapted for healthy controls (Niessen et al., 2017).

To this end, we first assessed the neural correlates of performance monitoring and error detection in – for a patient study – a relatively large sample of LH stroke patients (n = 24) by using classical ERP analyses (i.e., Ne/ERN and Pe). In addition, in a more explorative approach, we used more sensitive multivariate pattern classification analyses (MVPA) based on whole-brain activity. MVPA allows for the prediction, for instance, of a behavioral outcome (in our case, a correct or incorrect response) from specific patterns of the underlying brain activity (Bode et al., 2012, Bode and Stahl, 2014). For this, classifiers are trained on the spatially distributed ERP data across time, and it is analyzed how well these activity patterns predict correct from erroneous responses, providing an index for error-related information in the brain at each point in time. By comparing this prediction accuracy between stroke patients and controls, one can identify even small group differences in the temporal dynamics of cognitive processes related to performance monitoring.

The hypothesis that LH stroke patients would exhibit deficits in performance monitoring or error detection is based on the observation that the insula is a commonly damaged brain region after LH stroke. The insula has consistently been associated with error awareness in imaging studies with healthy participants as well as in patients with neurological or psychiatric diseases (for a review see Klein et al., 2013; Hester et al., 2009). Note that those patients included in the two previous studies showing no impairments in error detection for stroke patients (Stemmer et al., 2004, Maier et al., 2015) were not suffering from lesions affecting the insula.

## 2 Material and methods

### 2.1 Participants

**Fig 1:**
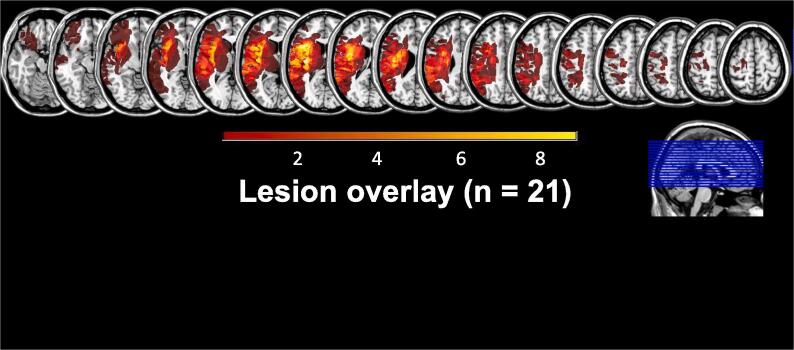
Lesion overlay. The lesion overlay for 21 of the 24 LH stroke patients is shown. One patient did not give informed consent to obtain the clinical CT/MRI images for lesion mapping, and for two patients, no clear lesion demarcation in early clinical imaging was detectable for lesion mapping despite persistent deficits. The overlay indicates that all patients suffered a single LH stroke affecting the left middle cerebral artery (MCA) territory. Color shades represent the increasing number of overlapping lesions. Slices are shown with MNI- coordinates ranging from −17 to 63.

Initially, 33 patients suffering from a single first-ever unilateral ischemic stroke affecting the left hemisphere and 33 healthy matched controls took part in the current study. The data from the healthy controls have been published previously (Niessen et al., 2017). In total, ten participants had to be excluded from subsequent analyses due to a variety of reasons: technical problems during EEG measurements (one control participant), violation of inclusion criteria (one patient with a visual field defect due to a stroke in the territory of the posterior cerebral artery; in one patient the initial clinical stroke diagnosis could not be confirmed by later examinations), and poor task performance (four patients conducted only undetected errors, meaning that they might not have understood the task and did not engage in performance monitoring; three patients revealed signs of exhaustion during testing). Hence, the final sample comprised 24 patients with LH stroke (mean age ± standard deviation [SD]: 56.4 ± 12.5 years, 4 female) and 32 healthy control participants (mean age ± SD: 56.4 ± 10.1 years, 12 female) who were matched for age (independent-samples *t*-test *p* = .99) and sex (Fisher’s exact test *p* > .1). MRIcron (Version 12, 2012; Rorden and Brett, 2000) was used for lesion mapping and estimating lesion size. Fig. 1 shows an overlay plot of the lesions of 21 out of the 24 patients (two patients had no demarcated stroke despite persistent deficit; one patient did not approve to obtain the clinical images). The left MCA territory was affected in all patients, and the most substantial lesion overlap was in the insula.

The study was performed following the Code of Ethics of the World Medical Association (Declaration of Helsinki) and approved by the local ethics committee of the Medical Faculty of the University of Cologne. After giving written informed consent, patients and controls were screened for right-handedness with the Edinburgh Handedness Inventory (EHI, Oldfield, 1971), and intact color vision with Ishihara color plates (Ishihara, 1994). Further, any signs of general cognitive decline as assessed with the Mini Mental State Examination (MMSE; Folstein et al., 1975) and the clock test (Shulman et al., 1993), as well as conspicuous symptoms of depression (Beck's Depression Inventory, BDI; Hautzinger, 1991) led to exclusion of participants. Patients who received a craniectomy during treatment of their stroke also had to be excluded due to potential EEG artifacts. Finally, patients were excluded for any sign of psychiatric disorders, including alcohol or drug abuse.

Patients were recruited prospectively during the sub-acute or chronic phase after stroke (i.e., more than four days post-stroke) resulting in a mean interval between testing and stroke of 54 days (SD: ± 65 days; range: 11–268 days).

A structured interview was additionally conducted with healthy control participants to assure no previous history of any neurological or psychiatric diseases. During the interview, the experimenter explicitly ruled out previously experienced (symptoms of a) stroke or transient ischemic attack (TIA). Table 1 summarizes the data of the stroke patients and controls.

**Table 1:** Data of stroke patients and healthy matched controls.

	Patients	Controls
N	24	32
Age (mean ± SD)	56.4 ± 12.5 years	56.4 ± 10.1 years
Age range	30–84 years	30–72 years
Sex	20 men, 4 women	20 men, 12 women
Clinical measures		
Days after stroke	54 ± 65 (range 11–268 days)	
ACL-k (max. 40, cut-off ≤ 33)	27.8 ± 10.3	
KAS (max. 80, cut-off for apraxia ≤ 76)	72.0 ± 12.3	
MRC Scale	4.7 ± 0.5	
Modified Rankin Scale	1.5 ± 1.2	

Table reports means and standard deviations (SD). ACL-k = short aphasia check list; KAS = Cologne Apraxia Screening; MRC = Medical Research Council scale.

### 2.2 Assessments and procedure

The complete data acquisition was carried out on one day in healthy controls, whereas the assessment was split and conducted on two days for stroke patients (Day 1: neuropsychological testing, Day 2: EEG study) to prevent fatigue effects during the primary task of interest. Data acquisition began with the neuropsychological tests related to the exclusion criteria (EHI, Ishihara, MMSE, clock test, BDI). The trail making test (TMT; Reitan, 1958) was used as a measure for executive functions and was always carried out as a paper and pencil test with the non-dominant, left hand, which was also the less affected ipsi-lesional hand in our left hemisphere stroke patients. For the patient group, additional neuropsychological tests were applied to assess clinical symptomatology: These comprised the Cologne Apraxia Screening (KAS; Weiss et al., 2013), the short version of the Aphasia Check List (ACL-k; Kalbe et al., 2005), the Medical Research Council scale for degree of paresis (MRC; Masur, 2000, O'Brien, 2010), and the modified Rankin scale for general impairment (e.g., degree of disability or dependence on caregiver) after stroke (mRS; Rankin, 1957).

**Fig 2:**
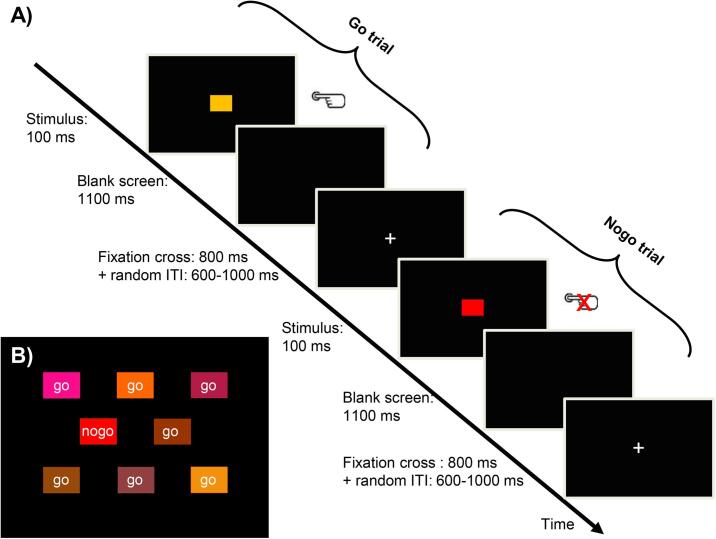
Go/Nogo task. A) The procedure for go and nogo trials was identical, apart from the instruction to press a button after the presentation of go-stimuli and to withhold a response after nogo-stimuli. Stimuli were presented for 100 ms, followed by a blank screen for 1100 ms. The appearance of a fixation cross signaled that participants were allowed to press the response button a second time in the case of detected errors. A random inter-trial interval (ITI) of 600–1000 ms was used, during which the fixation cross was presented as well. B) Illustrations of go- and nogo-stimuli (for details on RGB values of the different colors, see Niessen et al., 2017).

The main task implemented a previously established Go/Nogo paradigm (Niessen et al., 2017) with simultaneous electroencephalography (EEG) recordings. We decided to use the Go/Nogo task (which is one of the standard tasks for this type of research), because the error detection for inhibition errors (‘Did I press the response button or not?’) appears to be more feasible for stroke patients than error detection in more complex response conflict errors (‘Did I press the left or right response button?’), which is a feature of most other tasks (e.g., Erikson Flanker task or Stroop task). The nogo-stimulus was shining red, while seven reddish colors served as go-stimuli, with a go/nogo ratio of 80:20 (see Fig. 2b). Participants were asked to press a response button (LumiTouch, Burnaby, Canada) with the index finger of their non-dominant, left hand (i.e., the ipsilesional, non-paretic hand of patients) after the presentation of go-stimuli. After an incorrect button press in response to a nogo-stimulus, participants were trained to indicate the detection of these errors by pressing the response button a second time. The use of only one response button and implementing task difficulty through color discrimination (leading to an error rate of ~20%) was most convenient for the group of stroke patients. Pilot work with different task versions suggested that, for example, multiple response buttons or more complex rules for signaling error detection exhausted the stroke patients. In total, 360 trials separated into six blocks were presented using Presentation software (Neurobehavioural Systems, version 14.5). Each trial lasted 2000 ms and was otherwise identical for go and nogo trials (100 ms stimulus presentation, 1100 ms blank screen, 800 ms central fixation cross). Together with a randomized inter-trial interval between 600 and 1000 ms, the total task duration was approximately 20 min (see Fig. 2a). The six blocks were divided by breaks without predefined duration so that the breaks could be adjusted individually to the needs of each participant. All participants were asked to press the response button as fast as possible without mentioning accuracy. Signaling of error detection should occur after the appearance of the fixation cross (i.e., during the interval from 1200 to 2000 ms after stimulus onset). We used delayed signaling to preclude any confound, which could be caused by an overlap of ERP signals evoked by the second button press in the case of detected errors (e.g., an additional readiness potential) (Colebatch, 2007). Thus, for incorrect nogo trials, we differentiated between trials with two button presses and one button press, which from now on will be labeled as a detected and undetected error, respectively. Each participant underwent a training session (without EEG), including feedback about accuracy before the actual task, ascertaining that all colours were perceived correctly, and familiarizing the participant with the signalling procedure (for more details of the task, see Niessen et al., 2017). For both the healthy control and the stroke group, the experimenter then prepared the EEG cap, followed by the execution of the Go/Nogo task with simultaneous EEG recordings. As pointed out above, we wanted to prevent any performance impairment in stroke patients due to fatigue. Therefore, we conducted the training session and the experimental Go/Nogo task with the stroke patients on a second testing day.

### 2.3 EEG recording

EEG was recorded with a 64-channel system consisting of sintered Ag/AgCl electrodes (Acticap, Brain Products, Germany). The international 10–20 system served as the basis for positioning 61 electrodes on electrode caps. The reference and ground electrodes were arranged on the left mastoid and AFz, respectively. Vertical and horizontal electro-oculograms (EOG) were recorded from electrodes positioned below and above the left eye and on the outer left and right canthi. Data were recorded with a digital BrainAmp amplifier and VisionRecorder software (Brain Products, Germany). The sampling rate was 500 Hz. For online filtering, we employed a high-cutoff filter (70 Hz) and a notch filter (50 Hz).

### 2.4 Data and statistical analysis

Based on previous and our work, we used a minimum of six trials per response type as an individual inclusion criterion for ERP analyses (Niessen et al., 2017, Pontifex et al., 2010, Steele et al., 2016). As a consequence, we will present results from response rates and stimulus-locked ERPs from the complete sample, whereas reaction time (RT) analyses and response-locked ERP analyses were only conducted with a subgroup of participants with at least six (detected) error trials. The subgroup consisted of 17 patients (aged 53.6 ± 12.2 y, 13 male) and 24 healthy controls (aged 56.0 ± 9.4 y, 15 male). The minimum amount of trials necessary to conduct MVPA analyses was ten error trials (in order for standard cross-validation methods to be applied; see Bode and Stahl, 2014). In order to carry out this advanced analysis method, we created a second subgroup of patients and controls for the MVPA analysis consisting of 14 (out of 24) stroke patients (aged 54.9 ± 13.9 y, 11 male) and 21 (out of 32) controls (aged 56.3 ± 10.3 y, 13 male). For these participants, we collapsed data from detected and undetected errors to increase the statistical power for the MVPA analyses. Furthermore, due to the small number of incorrect go trials (i.e., correct responses after go-stimuli with subsequent error-signaling), misses, and undetected errors, we will only report response rates for these response types, but they will not be part of the electrophysiological analysis.

All statistical tests were Bonferroni-corrected if applicable. The Greenhouse-Geisser correction was applied when sphericity was violated, and alpha was adjusted accordingly. In addition to frequentist repeated-measures analysis of variances (RM ANOVAs), we also used Bayesian RM ANOVAs and non-directional independent-samples *t*-tests with a Cauchy prior of 0.707 (JASP version 0.9.0.1). We chose to report the Bayes factor and not the more commonly used effect sizes because, for our main results, effect sizes will be less relevant than Bayes factors in the context of non-significant group differences. For the analyses, we present two types of Bayes factors (BF): BF_10_ represents evidence for H1, which indicates that data are likely to stem from two different conditions/ groups rather than from the same condition/ group. In case that BF_10_ was smaller than 3, we report BF_01_ demonstrating evidence for H0 in favor over H1. For both cases (thus independent of being in favor of H0 or H1), a BF of 1–3 indicates anecdotal evidence, 3–10 moderate evidence, 10–30 strong, and more than 30 presents very strong evidence (Wagenmakers et al., 2016).

#### 2.4.1 Behavioral Data: Neuropsychological assessment and Go/Nogo task

The individual median RTs for detected errors after nogo-stimuli and for correct responses after go-stimuli were computed for the subset of 17 S patients and 24 controls. Post-error behavioral adjustments were operationalized as median RTs on trials following errors (post-error slowing; PES) minus median RTs on trials following correct trials (post-correct slowing; PCS) (Rabbitt, 1966) and will be referred to as ΔPES. The RT of the second button presses were only investigated to ensure that participants were responding within the delay interval and will not be discussed further. The mean of the individual median RTs was then computed, and relative response rates were calculated for go- and nogo-stimuli separately. Finally, we computed an error detection rate, which is the percentage of all reported errors relative to all errors made on nogo trials. All task-related variables (RTs of correct responses and detected errors, RTs reflecting ΔPES, error rate, and error detection rate) were submitted to an independent-samples *t*-test assessing potential group differences.

Time to completion in the Trail Making Test (TMT) was compared between groups with independent-sample *t*-tests for TMT Part A, TMT Part B, and the quotient thereof. Further, descriptive statistics of patients’ clinical scores are presented (see Table 1).

#### 2.4.2 Electrophysiological data

Pre-processing of the electrophysiological data and analysis on ERPs were conducted with BrainVision Analyzer 2. We applied the current source density transformations (Perrin et al., 1989) before conducting the ERP analyses because those transformations provide a superior spatial resolution in contrast to raw data (e.g., Stahl, 2010), and the transformed signals are independent of the mastoid references.

##### 2.4.2.1 Stimulus-locked ERPs

We were not only interested in response-locked ERPs, but we also wanted to compare neural activity associated with cognitive processes *before* error commission reflecting the neural input fed into the performance monitoring system. For that, we analyzed two classical ERP components associated with stimulus processing, namely the N2 and the P3. These stimulus-locked ERPs of correct responses to go-stimuli and correct withholds after nogo-stimuli were examined for all participants. The associated epochs for those two response types ranged from 100 ms before and 800 ms after stimulus onset. The interval of 100 ms preceding stimulus-onset was used for baseline correction. All data were screened for artifacts; trials exceeding minimum or maximum amplitudes of ±150 μV were rejected from further analyses. Ocular corrections were performed before artifact rejection (Gratton et al., 1983). The electrode site of interest was the centrally located FCz, which has been suggested to capture the inhibitory N2 (Enriquez-Geppert et al., 2010) and the attention-related P3a (Polich, 2007). Amplitudes and latencies of the N2 and P3 components were extracted at the most negative and positive peak in a time window from 200 to 350 ms and from 350 to 500 ms, respectively.

We compared the peak amplitudes and latencies of two components (N2, P3) by employing separate RM ANOVAs with the between-subjects factor *group* and the within-subject factor *response type* (correct responses and correct withholds for stimulus-locked data; correct responses and detected errors for response-locked data).

##### 2.4.2.2 Response-locked ERPs

For 17 patients^1^ and 24 healthy controls, ERPs for the two response types correct responses after go-stimuli and detected errors^2^ after nogo-stimuli were averaged time-locked to the onset of the response. These response types were evaluated in epochs that ranged from 100 ms before and 600 ms after a response occurred. Pre-processing of the response-locked ERPs regarding baseline correction, artifact rejection, and ocular correction were performed as described for the stimulus-locked ERPs. Peak amplitudes were extracted from each participant's grand average separately for both response types from the electrode sites FCz and Cz, since they best represented the Ne/ERN and Pe, respectively. Ne/ERN peak amplitude and latency (CRN for correct responses) were quantified as the most negative peak in the interval from 0 to 150 ms after response onset. Pe peak amplitude (Pc for correct responses) was quantified as the most positive peak in the time window from 150 to 300 ms after response onset.

Statistical analyses were performed as described for the stimulus-locked ERPs by using peak amplitudes and latencies of the two response-locked components Ne/ERN and Pe for errors and CRN and Pc for correct responses.

##### 2.4.2.3 Additional analyses

To ensure that our findings from response-locked data reflecting processes of performance monitoring were valid and robust, additional analyses of the area under the curve and peak-to-peak calculations were performed for response-locked ERPs for errors and correct responses. The area under the curve was extracted for the same time windows as had been used for peak extraction of amplitude and latencies from electrode FCz. Peak-to-peak was represented by the difference in amplitude and latency between Ne/ERN and Pe for errors (i.e., Ne/ERN amplitudes/ latencies were subtracted from Pe amplitudes/ latencies). Statistical comparisons were conducted employing RM ANOVAs with the factors group and response type for the area under the curve and peak-to-peak separately.

The area under the curve for the response types correct responses and errors were submitted to an RM ANOVA with the between-subject factor *group* and the within-subject factor *response type*. Peak-to-peak values were statistically compared with an independent-samples *t*-test.

##### 2.4.2.4 Multivariate pattern classification

For the multivariate pattern classification analysis (MVPA) of the response-locked data, we used the MATLAB-based Decision Decoding ToolBOX (DDTBOX; Bode et al., 2018) to investigate whether there were group differences between patients and controls in how well spatially distributed patterns of ERPs could be used to directly predict response types (i.e., errors and correct responses). In other words, we asked whether the underlying patterns of neural signals contained different amounts of error-related information across time between groups. For each participant separately, we trained a classifier to identify brain activity patterns for small analysis time windows (in particular leading up to the response onset; Bode and Stahl, 2014) to predict the response type in a given trial and averaged the resulting classification accuracy separately for each group. Then, we statistically tested the time course of classification accuracies between patients and controls using a series of *t*-tests for independent samples.

Data pre-processing for the response-locked analysis started with grouping correct responses and errors for each participant. To assure an identical amount of trials per classification group, the number of trials was matched to the response type with the fewest trials (the minimum amount of trials per response type was set to 10). The epochs of the response-locked data ranged from −100 to 300 ms, because we aimed to detect potential differences in classification accuracies before and after the response onset, given that these signals might be important neural precursors to post-response error-related processing (Bode and Stahl, 2014). Within these epochs, we constructed analysis windows of 10 ms width for a spatial decoding analysis (thus containing 5 data points per analysis time window) that were moved in non-overlapping 10 ms steps through the entire trial. Each analysis window contained data from all 61 EEG channels (but not the EOG channels), hence 61 × 5 = 305 data-points in total. Starting with the first analysis time window, a linear support vector machine classifier (standard regularization parameter C = 1) as implemented in the LIBSVM toolbox (Chang and Lin, 2011) was then trained on the data to define a decision boundary which optimally discriminated between activity patterns associated with the two examined response types (note that these analyses were conducted separately for each participant). For this, a “training set” consisted of 90% of trials randomly drawn from the overall data set. The remaining 10% of trials was used as an independent “test set” to explore whether the trained classifier could predict the corresponding response type from the EEG data. The resulting classification accuracy (i.e., how well this 10% of test data could be predicted above chance level) was noted, and the procedure was repeated in a classical ten-fold cross-validation procedure, which means that each subset of 10% of the data served as the test set once while the remaining 90% of data was used as the independent training set. To further prevent potential drawing biases, which could have occurred by allocating the data to the ten sets, the entire ten-fold cross-validation process was additionally repeated ten times (i.e., the random allocation into ten data sets was independently repeated ten times), which led to 100 analyses per time point in total. The prediction accuracies of these 100 analyses were then averaged, leaving one classification accuracy score per time window per participant (see Bode et al., 2018, for details on the classification procedure). The group averages of these very conservatively estimated classification accuracies could then be computed and statistically compared between patients and controls for each time point.

For comparisons of the classification accuracies for each analysis time window with chance level, a shuffled-labels analysis was conducted as implemented in DDTBOX. In short, an identical ten-fold cross-validation analysis with ten iterations was computed for each analysis time window and each participant, precisely as described for the main analysis, using the same data and “labels” for response types. The difference, however, was that the assignment of data (i.e., trials) to labels (i.e., trial type) was randomly shuffled for each cross-validation step (see Bode et al., 2018, Bode and Stahl, 2014, for details). The result is an empirical (control) distribution of classification results for the case that the association between data and response types were truly random. Paired-samples *t*-tests were calculated for each time point for each group separately to test for differences in classification accuracy between real and shuffled label classification results to determine when error-related information was present above chance. The next and crucial statistical test then involved again using independent-samples *t*-tests to test for a difference in (real) classification accuracies (i.e., error-related information) between patient and control groups. The adjusted critical alpha to correct for multiple comparisons was p = .00125 for this analysis (corrected for 40 time points).

#### 2.4.3 Influence of clinical and cognitive profile

Since a secondary interest of this study was to investigate a potential influence of stroke-related deficits on performance monitoring, we computed Pearson correlations between clinical measures and task-related variables for the patient group. We further applied linear regression analyses to further investigate the variables that showed significant correlations. We used one simple forward regression to predict the latency of ΔNe/ERN (dependent variable) based on lesion size (independent variable). To correct for multiple comparisons, the adjusted critical alpha level for the correlation analyses was p = .025.

We used lesion size and days post-stroke, and correlated those with behavioral (RT, error rate, and error detection rate) as well as neural measures of the Go/Nogo task. For the interested reader, the same correlational analysis can be found for the influence of apraxia and aphasia strength in the Supplementary Analysis 2. For the neural variables, we aimed to obtain information about neural indices independent of the individual baseline activity. We, therefore, computed difference scores for the stimulus-locked (ΔN2 and ΔP3 = correct withholds – correct responses) and response-locked ERPs (ΔNe/ERN and ΔPe = errors – correct responses) and used those for the correlation analysis. As a wide range of days post-stroke was present in our final patient sample, we additionally categorized our patients as sub-acute when they had suffered their stroke within the past 4–28 days (i.e., within 4 weeks post-stroke) and as being in their chronic phase thereafter (i.e., more than 4 weeks post-stroke). In addition to the correlational approach, we tested the influence of time after stroke on the above mentioned behavioural and neural measures by applying a two-samples *t*-test to the two sub-groups of sub-acute versus chronic patients.

## 3 Results

Unless stated otherwise, all results are expressed as the mean ± standard error of the mean (SEM).

### 3.1 Behavior

*Neuropsychological assessment.* According to the KAS (max. 80 points, cut-off for apraxia ≤76), 11 out of 24 patients showed symptoms of apraxia (72 ± 3 points, range: 24 to 80). Out of our 24 patients (28 ± 2 points, range: 5 to 40), fifteen patients were aphasic as indicated by the ACL-k (max. 40 points, cut-off ≤33). Of those, seven patients were suffering from expressive aphasia only, while eight patients had expressive and receptive language deficits. The degree of paresis of the contralesional hand (4.7 ± 0.1 points on the MRC scale: perfect/ unaffected score = 5) and the general impairment after stroke (1.5 ± 0.3 points of the mRS: perfect/ unaffected score = 0, max. impairment = 5) were mild to moderate. Thus, the current sample of left hemisphere stroke patients suffered from remarkable apraxic and aphasic deficits, whereas their deficits were less pronounced regarding hemiparesis and general impairment.

Further, the current LH stroke patients exhibited severe deficits in all domains of executive control assessed by the TMT. Five participants were not able to complete part B of the TMT in the available time. Compared to healthy controls, stroke patients were generally slower [TMT Part A: patients 48 ± 6 s, controls 29 ± 2 s; *t*(54) = 3.19, *p* < .01, BF_10_ = 38] and were more vulnerable to interference [TMT Part B: patients 132 ± 15 s, controls 64 ± 5 s; *t*(49) = 4.15, *p* < .001, BF_10_ = 1563]. Correcting for motor speed (which is supposed to be measured with Part A), the TMT quotient reflecting relative interference was still significantly higher for patients than controls [TMT quotient: patients 3.6 ± 0.4 s, controls 2.3 ± 0.1 s; *t*(49) = 3.30, *p* < .01, BF_10_ = 132].

**Fig 3:**
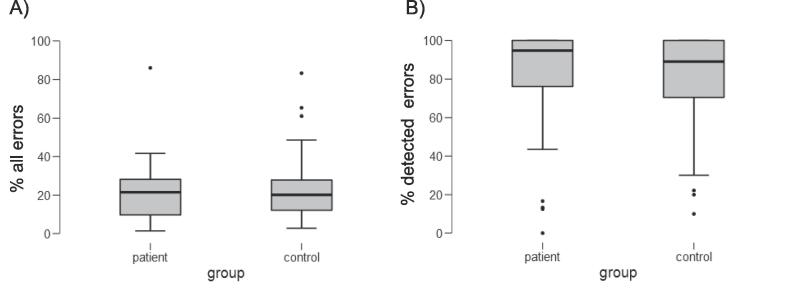
Behavioral results for error rate and error detection rate. A) The boxplot shows the percentage of all errors made after nogo-stimuli for patients and controls separately, that were significantly different from each other. B) The percentage of all detected errors amongst all errors made after nogo trials is displayed, again showing no group difference. For both boxplots, the thick line indicates the group median and error bars represent 95% confidence intervals.

*Behavioral parameters of the Go/Nogo task*^3^*.* For the Go/Nogo task, response rates were evaluated as relative percentages separately for each stimulus type. On go trials, the majority of responses were correct for both patients (95.0 ± 2.0%) and controls (95.0 ± 0.9%). Hardly any incorrect go trials were observed for patients (0.9 ± 0.3%) and for controls (0.6 ± 0.1%), while misses after go trials occurred more often, but similarly in patients (4.1 ± 1.7%) and controls (4.8 ± 0.9%). Group differences could not be found for any of these response types [*t*(54) = 0.19, *p* = .85, BF_01_ = 3.6; *t*(54) = 0.91, *p* = .37, BF_01_ = 2.4; and *t*(54) = −0.38, *p* = .71, BF_01_ = 3.5, respectively]. Compared to go trials, the general accuracy on nogo trials was lower, but similar for stroke patients (22.1 ± 3.5% overall errors) and healthy controls [24.3 ± 3.3%; *t*(54) =  −0.46, *p* = .65, BF_01_ = 3.4]. The error detection rate for errors after nogo-stimuli, i.e., the relative amount of detected errors, was again similar for both patients (77.8 ± 6.9% detected errors) and controls [78.3 ± 4.8%; *t*(54) =  −0.07, *p* = .94, BF_01_ = 3.7; see Fig. 3 and Table 2]. Additional explorative analyses revealed that the detection of conducted errors became generally better with time on task, which was independent of group (but please see Supplementary Fig. S1 for more details).

**Table 2:** Overview of behavioral data.

	Patients	Controls	*t*-tests significance
**Analysis of Trail Making Test**	N = 24	N = 32	
TMT A	48.3 ± 5.9	28.6 ± 1.9	p < .01
TMT B	131.6 ± 15.3	64.2 ± 5.5	p < .001
**Analysis of response types**	N = 24	N = 32	
Incorrect go trials (in %)	0.9 ± 0.3	0.6 ± 0.1	p = .55
Misses on go trials (in %)	4.1 ± 1.7	4.8 ± 0.9	p = .75
All errors on nogo trials (in %)	22.1 ± 3.5	24.3 ± 3.3	p = .54
Detected errors of all nogo errors (in %)	77.8 ± 6.9	78.3 ± 4.8	p = .42
**Analyses of reaction times**	N = 17	N = 24	
Baseline RT (go correct)	433 ± 15 ms	416 ± 15 ms	p = .46
RT for detected errors	352 ± 16 ms	350 ± 16 ms	p = .96
ΔPES after detected errors	65 ± 18 ms	86 ± 19 ms	p = .44

Table reports means and standard error of the mean (SEM). TMT = Trail Making Test.

Regarding RT, there was no significant group difference for correct responses after go stimuli, from now on referred to as ‘baseline RT’ [patients: 433 ± 15 ms vs. controls: 416 ± 15 ms; *t*(39) = 0.75, *p* = .46, BF_01_ = 2.6]. RTs of errors were shorter than RTs of correct responses, but the mean RT was again similar for patients (352 ± 16 ms) and controls [350 ± 16 ms; *t*(39) = 0.05, *p* = .96, BF_01_ = 3.2]. Finally, PES could be observed for both groups, with a similar slowing of responses in trials following errors for both groups [ΔPES; patients: 65 ± 18 ms; controls: 86 ± 19 ms; *t*(38) = −0.77, *p* = .45, BF_01_ = 2.5]. Thus, we can conclude that at the behavioral level, stroke patients were able to perform the Go/Nogo task as well as healthy controls, despite persistent deficits (e.g., apraxia, aphasia, and executive functions).

### 3.2 Electrophysiological data

#### 3.2.1 Stimulus-locked ERPs

The RM ANOVA for N2 amplitudes for correct go and correct nogo trials showed a significant main effect of response type [*F*(1, 53) = 8.05, *p* < .01, BF_10_ = 9.6], but neither a significant main effect of group [patients: −21.5 ± 3.2 μV/m^2^, controls –22.5 ± 2.7 μV/m^2^; *F*(1, 53) = 0.06, *p* = .81, BF_01_ = 2.1] nor a significant interaction [*F*(1, 53) = 1.49, *p* = .23, BF_01_ = 1.9]. Thus, independent of group, the N2 amplitude was generally larger following nogo-stimuli (–23.7 ± 2.2 μV/m^2^) than go-stimuli (−20.3 ± 2.0 μV/m^2^). When examining N2 latency, the main effect of response type [*F*(1, 53) = 0.37, *p* = .55, BF_01_ = 3.7] was not significant, while a significant main effect of group emerged [*F*(1, 53) = 7.49, *p* < .01, BF_10_ = 5.4]. This suggests that processing was slower for patients (303 ± 6 ms) than for controls (280 ± 5 ms). The interaction was not significant [*F*(1, 53) = 1.24, *p* = .27, BF_01_ = 2.2].

**Fig 4:**
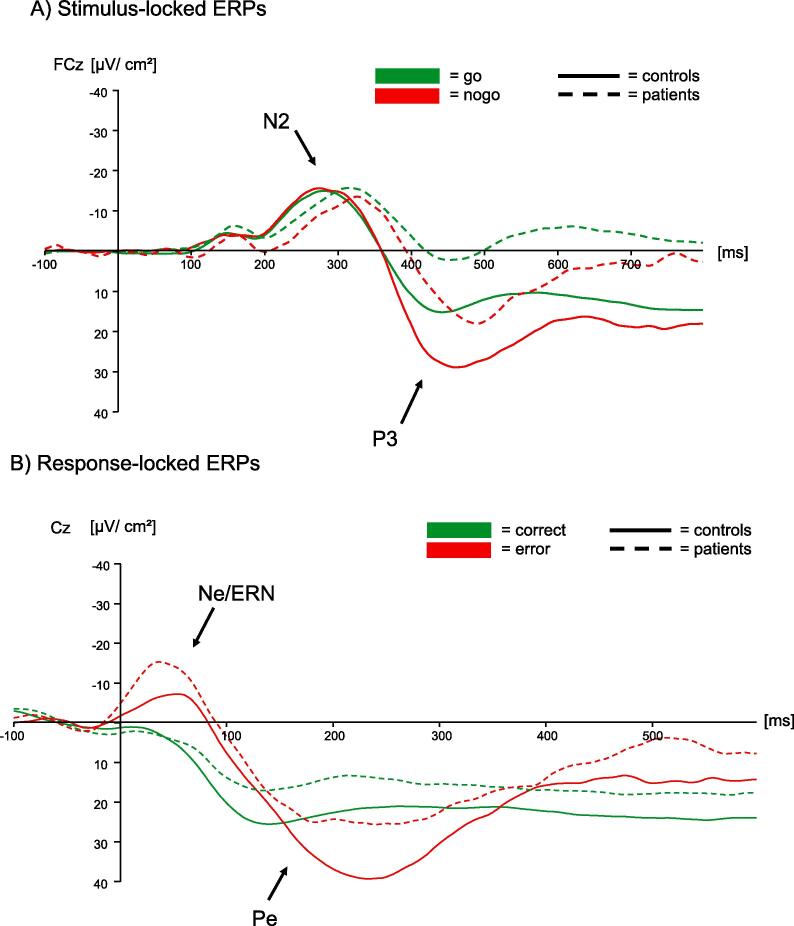
Main results of stimulus- and response-locked ERPs A) The N2 and P3 components are shown for correct responses to go stimuli (green) and correct withholds after nogo-stimuli (red) for all participants. Dashed lines represent the patient group, and the bold line represents healthy controls. Independent of response type, the N2 peaked later in patients than in controls. The P3, being larger in amplitude for nogo than go trials, was smaller in amplitude for patients than for controls. B) For all participants who made at least six detected errors (17 patients and 24 controls), the Ne/ERN and Pe components are illustrated for correct responses after go-stimuli (green) and detected errors (red). The Ne/ERN was larger for errors than for correct responses, but there was no significant difference between the groups. The same was true for the Pe component for which again no significant group difference emerged. For illustrative purposes only, results are plotted using a high cut-off filter (10 Hz, 12 dB/oct). (For interpretation of the references to color in this figure legend, the reader is referred to the web version of this article.)

Analysis of the P3 amplitudes revealed two significant main effects. The main effect of response type [*F*(1, 53) = 36.69, *p* < .001, BF_10_ = 166373] indicated that the P3 amplitude was generally larger following nogo- than go-stimuli (24.8 ± 2.8 μV/m^2^ and 11.3 ± 1.9 μV/m^2^, respectively). The main effect of group [*F*(1, 53) = 18.89, *p* < .001, BF_10_ = 326] revealed that independent of response type, stroke patients had a lower P3 amplitude compared to controls (9.0 ± 3.2 μV/m^2^ and 27.1 ± 2.7 μV/m^2^, respectively). The interaction between response type and group was not significant [*F*(1, 53) = 0.47, *p* = .49, BF_01_ = 3.0]. Regarding P3 latency, we only observed a significant main effect of response type [*F*(1, 53) = 9.86, *p* < .01, BF_10_ = 15], while the main effect of group [patients: 453 ± 6 ms, controls: 453 ± 5 ms; *F*(1, 53) = 0.01, *p* = .96, BF_01_ = 3.5] and the interaction effect [*F*(1, 53) = 0.02, *p* = .89, BF_01_ = 3.9] were not significant. The main effect of response type demonstrated a faster processing of go-stimuli (445 ± 5 ms) in contrast to nogo-stimuli (460 ± 4 ms). The results of N2 and P3 components are depicted in Fig. 4a.

#### 3.2.2 Response-locked ERPs

The RM ANOVA for Ne/ERN amplitudes on the respective subgroup of stroke patients (n = 17) and controls (n = 24) demonstrated a significant main effect of response type [*F*(1, 38) = 10.29, *p* < .01, BF_10_ = 23]. This was due to larger amplitude after errors (–23.5 ± 3.3 μV/m^2^) than after correct responses (−11.8 ± 1.6 μV/m^2^). However, we did not observe a main effect of group [*F*(1, 38) = 1.18, *p* = .29, BF_01_ = 2.6], hence similar amplitudes for patients (−19.7 ± 2.9 μV/m^2^) and controls (−15.6 ± 2.5 μV/m^2^), and no significant interaction effect [*F*(1, 38) = 1.31, *p* = .26, BF_01_ = 1.9]. Please see Table 3 for amplitude sizes of the Ne/ERN and CRN for both groups separately. Results for Ne/ERN latency were very similar: a significant main effect of response type [*F*(1, 38) = 10.24, *p* < .01, BF_10_ = 33] suggested faster processing of correct responses (36 ± 3 ms) compared to errors (51 ± 4 ms) for both stroke patients and healthy controls. The main effect of group [patients: 46 ± 4 ms, controls: 42 ± 4 ms; *F*(1, 38) = 0.43, *p* = .52, BF_01_ = 3.1] and the interaction [*F*(1, 38) = 0.17, *p* = .68, BF_01_ = 3.1] were again non-significant.

**Table 3:** Peak amplitudes of response-locked ERPs for correct and error trials.

	Patients	Controls
CRN [µV]	−11.8 ± 1.9	−12.0 ± 2.3
Ne/ERN [µV]	−27.7 ± 5.9	−19.4 ± 3.8
Pc [µV]	20.3 ± 4.0	27.0 ± 4.1
Pe [µV]	39.5 ± 5.9	48.1 ± 6.7

The table reports means and standard error of the mean (SEM). Please note that there was no significant difference between the groups.

Regarding Pe amplitudes, we observed a significant main effect of response type [*F*(1, 38) = 21.20, *p* < .001, BF_10_ = 959], while the main effect of group [patients: 30.0 ± 5.0 μV/m^2^, controls 38.0 ± 4.2 μV/m^2^; *F*(1, 38) = 1.56, *p* = .22, BF_01_ = 1.9] and the interaction between response type and group [*F*(1, 38) = 0.01, *p* = .92, BF_01_ = 2.5] were not significant. The main effect of response type suggested larger Pe amplitudes after errors (43.8 ± 4.6 μV/m^2^) than after correct responses (24.1 ± 3.0 μV/m^2^) which was similar for patients and controls. Finally, for Pe latency we did not observe any significant differences between response types [*F*(1, 38) = 2.54, *p* = .12, BF_01_ = 1.2], groups [patients: 218 ± 9 ms, controls: 229 ± 8 ms; *F*(1, 38) = 0.83, *p* = .37, BF_01_ = 2.5], and also no significant interaction between response type and group [*F*(1, 38) = 0.05, *p* = .83, BF_01_ = 3.1]. The results of Ne/ERN and Pe components are shown in Fig. 4b.

#### 3.2.3 Additional analyses on response-locked data

Given that the previous group comparisons for the response-locked ERPs were non-significant, we conducted additional analyses using the area under the curve and peak-to-peak as alternative measures to make sure that we did not overlook any differences that were due to the choice of analysis techniques. However, there were again no significant differences between groups for area under the curve of the Ne/ERN [patients: 13.7 ± 1.6, controls: 15.1 ± 1.4; *F*(1, 38) = 0.40, *p* = .53, BF_01_ = 3.0] and the Pe [patients: 22.0 ± 3.8, controls: 31.9 ± 3.2; *F*(1, 38) = 3.93, *p* = .06, BF_01_ = 0.8]. The interactions between group and response type were also not significant [Ne/ERN: *F*(1, 38) = 1.27, *p* = .27, BF_01_ = 1.5, Pe: *F*(1, 38) = 0.68, *p* = .42, BF_01_ = 2.3, respectively]. This pattern was further supported by the results of the peak-to-peak analyses. We did not detect any significant group difference regarding build-up from Ne/ERN to Pe component in contrast to the build-up from CRN to PC, neither in terms of amplitude [patients: −18.6 ± 3.6 µV, controls: −17.4 ± 3.1 µV; *F*(1, 38) = 0.08, *p* = .79, BF_01_ = 3.9] nor in terms of latency [patients: 187 ± 9 ms, controls: 172 ± 8 ms; *F*(1, 38) = 1.42, *p* = .24, BF_01_ = 3.1]. Again, the interaction terms between group and response type were also not significant [amplitude: *F*(1, 38) = 1.40, *p* = .25, BF_01_ = 0.7, latency: *F*(1, 38) = 0.08, *p* = .78, BF_01_ = 0.3, respectively].

Hence, irrespective of analysis method, all our results suggested that stroke patients showed a highly similar pattern of results in the Go/Nogo task compared to healthy controls.^4^

#### 3.2.4 Multivariate pattern analysis

**Fig. 5:**
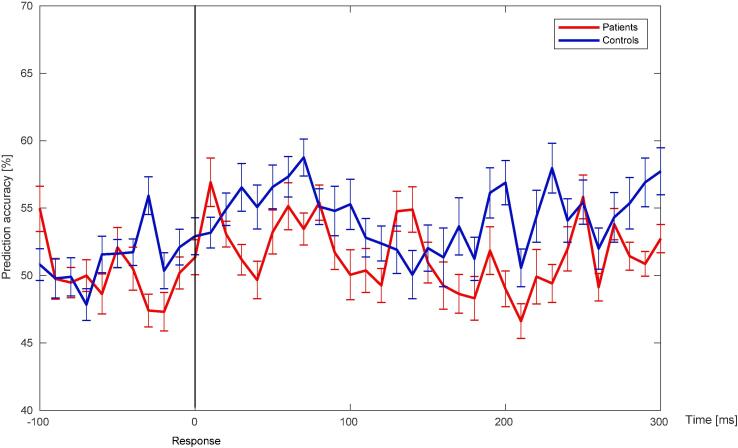
Main results of response-locked MVPA. Results of the MVPA on response-locked data are illustrated as the percentage of classification accuracy (i.e., indicating how well errors and correct responses could be predicted from small time windows of spatially distributed brain activity patterns). At time 0 (‘Response’), the (correct or incorrect) button press occurred. In this illustration, the x-axis represents the chance level. Mean accuracies are shown for patients (red line) and controls (blue line) separately, with error bars indicating standard errors of the mean. Classification accuracies did not differ between groups for any of the tested time windows. (For interpretation of the references to color in this figure legend, the reader is referred to the web version of this article.)

Highly sensitive MVPA was used to identify error-related information represented in distributed brain activity patterns for the different response types during the time intervals of interest. First, for all participants combined, we found a significant prediction of response type (correct vs error) in time windows immediately following responses (from 0 to 20 ms and 40–80 ms; all p < .05). These effects were expected, because the time window corresponds to the time window in which the Ne/ERN peaks. After demonstrating that information about the response type was present and decodable in the EEG data, we compared the classification accuracies of stroke patients and healthy controls within these time windows. Crucially, we did not find any difference for patients and controls in any of these time windows (all p > .14, see Fig. 5). In summary, the classifier showed similar performance for both groups, indicating that the underlying brain activity in patients and controls was similarly suited to enable the classifier to discriminate correct responses and error responses in response-locked brain activity.

### 3.3 Influence of clinical and cognitive profile

The time interval post-stroke (in days) did not significantly correlate with any task-related measure (all *p* > .05; note Type I error-corrected *p* level = 0.025). For lesion size, we observed a significant association with the latency of the ΔNe/ERN (*r* = 0.63, *p* = .009) indicating that patients with large lesion sizes needed longer to detect an error (for all other correlations *p* > .025). Linear regression analyses confirmed results from the correlation analyses. The regression of the latency of the ΔNe/ERN by lesion size was significant (F(1,12) = 9.50, p < .01, r^2^ = 0.44).

For brevity, we here only report significant correlations, but please see Table 4 for a detailed summary of all correlational analyses.

**Table 4:** Results of correlational analysis.

	Lesion size	Days post stroke
Baseline RT	r = 0.23 p = .31	r = −0.37 p = .07
Error rate	r = −0.43 p = .05	r = 0.10 p = .63
Error detection rate	r = 0.30 p = .19	r = 0.11 p = .61
ΔN2 amplitude	r = 0.16 p = .48	r = −0.15 p = .47
ΔN2 latency	r = −0.10 p = .67	r = −0.29 p = .17
ΔP3 amplitude	r = 0.14 p = .54	r = 0.24 p = .26
ΔP3 latency	r = 0.20 p = .38	r = 0.03 p = .90
ΔNe/ERN amplitude	r = 0.12 p = .68	r = 0.41 p = .10
ΔNe/ERN latency	**r = 0.67** **p = .01**	r = 0.30 p = .24
ΔPe amplitude	r = −0.45 p = .11	r = −0.40 p = .11
ΔPe latency	r = −0.40 p = .16	r = −0.48 p = .05

Table shows the correlation coefficients and p-values of the corresponding variables. Coefficients printed in bold are significant according to the Type I error-corrected *p* level.

The sub-group analysis of patients in the sub-acute and chronic phase after stroke revealed a significant group difference for the ΔNe/ERN amplitude size only (t(15) = −2.33, p = .034; all other p > .29). Patients in their sub-acute phase showed no significant difference in the Ne/ERN amplitude for correct responses (−13.57 ± 1.79) and errors (−15.26 ± 7.75), while chronic patients showed the commonly observed pattern of increased Ne/ERN amplitudes for errors (−38.67 ± 7.15) as compared to correct responses (−10.14 ± 3.28).

## 4 Discussion

### 4.1 Summary of findings

Using a simplified color-Go/Nogo-task, we investigated the integrity of performance monitoring and error detection in a large sample of LH stroke patients. The left MCA territory, which is the most frequently damaged stroke territory (Bogousslavsky et al., 1988), was affected in all patients. As confirmed by neuropsychological tests, patients experienced deficits of motor cognition, language processing, and executive functions. Our electrophysiological data also indicated deficits in stimulus processing in the LH stroke patients compared to the controls: Patients were slower in the processing of the two stimulus types indicated by a later peaking of the N2. Further, the demand for more attentional resources related to stimulus-elicited conflict (reflected by the reduced P3/ P3a amplitude; Polich, 2007) was less distinct in patients. The N2 is commonly associated with processes of inhibition (Falkenstein et al., 1999) and conflict processing (Groom and Cragg, 2015). A delay in the initiation of stimulus processing, but also deficits with conflict detection and increased demand for attentional resources, could, therefore, hint at apparent, subthreshold difficulties of patients with the Go/Nogo task that were nevertheless not strong enough to result in overt performance deficits.

Intriguingly, we did not find any abnormalities in the behavioral markers of performance monitoring in the Go/Nogo task for the LH stroke patients despite their obvious clinical symptoms. Compared to healthy age-matched controls, patients showed both a similar error rate and a similar error detection rate. There was neither a general RT difference (i.e., for correct responses after go stimuli), nor a differential RT pattern for the two response types. Both groups were comparable, responding faster on error trials compared to correct trials, and both groups slowed down to a similar degree immediately after an error (i.e., similar PES). These findings indicate similar error detection performance and behavioral adjustments after errors in the two groups. This was surprising considering that the largest lesion overlap in our sample was located in the left insula. Previous studies suggested that the insula may constitute the neural correlate of anosognosia (Moro et al., 2011), which is defined as a diminished deficit awareness due to brain damage (Vossel et al., 2012). While anosognosia has classically been related to right hemisphere damage, recent reports showed that anosognosia also occurs for aphasia (Cocchini et al., 2009) and apraxia (Kusch et al., 2018) after left hemisphere stroke. Thus, given the conceptual similarities of error awareness and anosognosia, we expected to observe deficits in error awareness in our patient sample. Besides, the fact that LH stroke patients showed not even slower responses, in general, was particularly notable given that nearly half of the sample suffered from apraxia, and given that they were significantly slower in finishing the TMT (on average 20 s and 70 s slower on Part A and B, respectively). We also tested whether the severity of apraxia and aphasia influence the performance parameters and neural correlates related to the Go/Nogo task (Canzano et al., 2014, Kusch et al., 2018). We found longer RTs with stronger apraxia and aphasia, but interestingly no further influence on behavioural or neural measures related to error detection. We report and discuss these analyses in more detail in the Supplement (Supplementary Analysis 2).

The lack of significant differences in behavior between healthy control and stroke groups in our Go/Nogo task was surprising because several studies reported behavioral deficits in response tasks such as the Erikson flanker task and the Stroop task (e.g., Swick and Turken, 2002, Wessel et al., 2014). See Supplementary Table 2 for a direct comparison of all EEG studies testing error processing in stroke patients. These striking behavioral findings were further supported by the ERP analyses, which showed no group differences in neural correlates of performance monitoring and error processing (i.e., response-related ERP components, Ne/ERN, and Pe). To validate our findings, we used different ERP measures, yet no group difference could be identified. Even the very sensitive MVPA, which utilizes the entire spatially distributed activity across all scalp electrodes and detects subtle information that would otherwise be overlooked (e.g., Bode et al., 2012, Bode and Stahl, 2014), did not find any group differences in error-related information neither before nor after the response. This results remained even when a very liberal statistical threshold was applied. The information time course was well above chance level for both groups but highly similar, pointing to preserved performance monitoring in our patients.

Several new and important questions arise from our current findings: Why did our results not mirror previous findings? Why did we find a difference in early, stimulus-related but not in late, response-related indicators of cognitive processing? Moreover, what do the findings mean for the underlying basic performance monitoring processes of LH stroke patients?

### 4.2 Divergence of our findings from previous studies

Two critical factors that might have led to different – and presumably less biased – findings compared to previous studies are briefly discussed here.

One crucial improvement of our study was the standardization of the error signaling procedure for patients, which is necessary for establishing a valid association between error detection and the Pe component (Maier et al., 2015). Reported abnormalities of the Pe amplitude in some stroke patient studies might rely on the lack of assessing patients’ error detection (e.g., Ullsperger, 2006, Ullsperger et al., 2002, Wessel et al., 2014). Without an indicator of error detection, a reduced Pe amplitude in one group might reflect (hidden) group differences in error awareness. Since most previous experimental tasks did not capture this process explicitly (for an exception see Maier et al., 2015), reported “abnormalities” in Pe amplitudes have to be interpreted with caution. Importantly, our standardized approach confirmed the association between error awareness and the Pe amplitude and showed at both the behavioral and the neural level that this process was unaffected by LH stroke.

The second advantage of our task was the use of non-linguistic stimuli. To substantiate the importance of this factor, we want to draw attention to some inconsistencies in the recent literature: Whereas some studies also found similar error rates for patients and controls, other studies reported an increased amount of errors in patients (see Supplementary Table 2). Interestingly, all studies demonstrating elevated error rates in patients used linguistic stimuli (letters or words, Gehring and Knight, 2000, Swick and Turken, 2002, Wessel et al., 2014). Given that the majority of these patients suffered from LH stroke, the aphasic deficits common after LH stroke most likely impaired processing of linguistic stimuli and thereby led to increased error rates. Unfortunately, these studies reported no data concerning the assessment of aphasia. Thus, we can only speculate that elevated error rates of stroke patients might have been a result of stroke-related cognitive (here: aphasic) deficits rather than being related to performance monitoring *per se*.

More generally, however, the diverging findings might not only result from varying methods but could also stem from conceptual differences (e.g., action types, task difficulty). The (preserved) awareness of errors in *simple* actions (i.e., button presses) differs from the (impaired) error awareness for *complex* actions (i.e., performing gestures; Canzano et al., 2014, Howard et al., 2019, Kusch et al., 2018). Further, differences in task difficulty could explain different performance results in stroke patients and controls, if patients were pushed to the limit of their cognitive capacity (Remington and Loft, 2015). Finally, previous studies may have suffered from relatively low statistical power due to small sample sizes resulting in the inability to differentiate between the Ne/ERN and CRN in their patient groups (e.g., Ullsperger and Cramon, 2006). The current large patient sample allowed a clear differentiation between Ne/ERN and CRN. Moreover, the variance (e.g., of error rates; see Fig. 3) was similar between the current LH stroke patients and healthy controls. Thus, differences in variance cannot account for the lack of significant group differences.

### 4.3 Stroke-induced alterations in stimulus-related but not in response-related processing

The fact that we did not observe alterations of the *response*-related ERP components in stroke patients was especially striking, considering that both *stimulus*-related ERP components were abnormal in patients as compared to controls. The alterations of N2 and P3 imply that the presented stimuli were processed and evaluated less efficiently in LH stroke patients, and it is, therefore, reasonable to assume that this generated a different neural input for the performance monitoring system. Given that we did not observe any successive changes (neither concerning behavior nor response-locked processes), it follows that the observed deficits in stimulus processing and evaluation were successfully compensated in the LH stroke patients. It is widely accepted that under excessive task demands, a breakdown of performance can be observed in patients (Price and Friston, 1999). Simplifying the Go/Nogo task as much as we did allowed us to specifically test the cognitive functions of interest, i.e., performance monitoring and error processing/ detection in simple actions. More complex tasks also involve related cognitive functions such as working memory (Wessel et al., 2014), which was shown to impede error processing and error detection in particular (Maier and Steinhauser, 2017). Such interdependencies of multiple cognitive functions during a given task, as well as the separation of these functions, are frequent and challenging problems of studies investigating cognitive control in patients (Fellows, 2017) – a challenge that we successfully met with our current task. Hence, our findings suggest that even under circumstances in which stimulus processing is sub-optimal and demands more resources for stroke patients, the general performance monitoring system can be preserved.

### 4.4 Significance of findings for the processes underlying basic performance monitoring in LH stroke patients

In previous patient studies of error processing, patients were included based on their lesion location in order to elucidate the involvement of specific brain regions in performance monitoring processes (see Supplementary Table 2). In our study, the enrolled stroke patients were intended to represent the majority of patients suffering a left hemisphere stroke (Bogousslavsky et al., 1988), which we confirmed by clinical, neuropsychological, and imaging data. For this more representative group of LH stroke patients, we demonstrated using both standard and advanced analysis techniques that the *basic* performance monitoring was intact. Interestingly, the explorative analysis between the LH patients in their sub-acute and chronic phase after stroke revealed no difference between the Ne/ERN and CRN amplitude in sub-acute patients, while chronic patients showed the commonly observed pattern of increased Ne/ERN amplitudes compared to CRN amplitudes. This finding is in contrast with previous studies, which reported absent differences between the Ne/ERN and CRN in their patients, who were all in their chronic phase (e.g., Ullsperger and Cramon, 2006; Wessel et al., 2014). Due to the cross-sectional design of our study, and the relatively small sample sizes of both sub-groups, however, these results should be interpreted with care. One possible explanation for these results is that the stroke-related lesions in the left hemisphere initially disturbed the neural mechanisms of performance monitoring. Notably, in the sub-acute phase after stroke, this potential disturbance did not lead to behavioral impairments, which may result from a compensation by non-lesioned brain regions (ipsi- or contralesionally). Thus, during stroke rehabilitation, the neural mechanisms of performance monitoring might have been reinstated again, which would explain why in our chronic patients, the typical pattern of increased Ne/ERN amplitudes compared to CRN amplitudes were observed again. However, these possible explanations clearly need to be systematically addressed in longitudinal studies.

Another argument for the preserved basic performance monitoring in our patients could be that the brain regions lesioned in our patients were not involved in performance monitoring and error processing in a relevant manner. Indeed, no patient showed a lesion of the dACC, which has been suggested to generate the Ne/ERN. Importantly, this allowed us to observe a reliable Ne/ERN in the first place. However, it is interesting that we did not find any variation in this component, because besides the dACC, a large network is recognized as being involved in performance monitoring. This network is assumed to comprise the DLPFC, basal ganglia, thalamus and insula (Bruijn et al., 2011, Seifert et al., 2011, Ullsperger, 2006), of which several regions were lesioned in our patient sample. Despite this, our patients did not show an effect on basic performance monitoring nor the corresponding neural markers. This observation was especially surprising since even individual differences such as age (Hoffmann and Falkenstein, 2011), perfectionism (Stahl et al., 2015) or anxiety (Moser et al., 2013), which supposedly have smaller effects, were nevertheless related to variations in the Ne/ERN. This makes the preserved performance monitoring and unchanged neural correlates despite the striking cortical lesions in our patient sample even more remarkable.

### 4.5 Strengths and limitations

Although we suggest that our version of the Go/Nogo task and our general study design has many advantages, it also has disadvantages. A reduction of the total duration of the experiment was necessary to prevent fatigue effects in the patient group. Consequently, the small number of trials (in particular, the small number of nogo trials) reduced the power of the analyses. As a result, we were unable to analyze ERPs related to undetected errors. Although it is difficult to increase the rate of undetected errors specifically, this warrants particular attention in future studies. The fact that the experiment was conducted in one session for the healthy control group, while patients were tested on two days, could have led to stronger cognitive fatigue in controls compared to patients. However, we did not observe a differential decrement in performance for the two groups throughout the Go/Nogo task (see Supplementary Fig. S1). This observation, together with the fact that the task would have been naturally more demanding in patients than controls, makes it unlikely that this caused any significant differences in fatigue between the two groups. One potential disadvantage of our approach is that because a simplified version of the Go/Nogo task was used, we necessarily observed a relatively low number of errors, which in turn led to reduced statistical power. Whether our results hold in more complex tasks and more demanding environments is, therefore, a question for future research. Hence, future studies are warranted that implement experimental tasks with varying degrees of task difficulty.

Compared to prior studies, the relatively large sample size (n = 24; the largest previous sample comprised nine patients; Ullsperger and Cramon, 2006) allowed us to increase the statistical power. On the other hand, the sample size of 24 patients is still relatively small in comparison with most EEG studies using healthy participants. Another innovative aspect of our study was that we not only assessed stroke-related clinical and cognitive deficits (Maier et al., 2015, Stemmer et al., 2004) but also used this information to examine a potential relationship between the degree of deficits and performance monitoring. Finally, MVPA enabled us to investigate the temporal dynamics of error processing with the conclusion that not even the time courses of error-related information represented in the neural signals differed between LH stroke patients and controls. These findings have important implications for rehabilitation of LH stroke patients since this implies that it might not be necessary or advisable to specifically target performance monitoring with rehabilitative therapies for LH stroke patients. If deficits with error detection are apparent in individual patients, those might be due to interfering deficits in other cognitive domains (like language processing or working memory). This further implies that breaking down tasks into simple components might be helpful for stroke patients to monitor their performance successfully.

## 5 Conclusion

The current study investigated performance monitoring and error detection in patients with left hemisphere (LH) stroke by using an adapted color-discrimination Go/Nogo task that allowed the signaling of consciously detected errors while recording EEG. The group of LH stroke patients (n = 24) experienced clinically relevant cognitive deficits (i.e., aphasia and apraxia) and executive dysfunction. Despite these stroke-related deficits, we did not observe any behavioral or neural impairment related to performance monitoring and error processing: Patients showed similar RTs, PES, error rates, and error detection rates, as well as similar amplitudes and latencies of the Ne/ERN and Pe components. The absence of group differences in error-processing was confirmed by more sensitive MVPA analyses based on spatially distributed brain activity patterns. We demonstrated the validity and specificity of our findings by showing abnormalities in stimulus-processing for the patient group (i.e., N2 and P3 components). The current results stress the importance of well-designed experimental tasks adopted for patient studies that precisely tap on specific cognitive functions (here: performance monitoring and error processing) and are less affected by other cognitive processes (here: language processing and working memory) that might also be disturbed in the patients. Finally, future patient studies should also characterize the cognitive and clinical profile of the given patient sample to enable the investigation of potential relationships between the cognitive/ clinical deficits of the patients and specific measures of their task performance.

## 6 Funding source

10.13039/100001641GRF gratefully acknowledges support by the 10.13039/501100011566Marga and Walter Boll Foundation. SB is supported by an 10.13039/501100000923Australian Research Council grant (DP160103353).

## CRediT authorship contribution statement

**Eva Niessen:** Conceptualization, Software, Investigation, Data curation, Formal analysis, Writing - original draft, Visualization, Writing - review & editing. **Jana M. Ant:** Investigation, Data curation, Writing - review & editing. **Stefan Bode:** Software, Writing - review & editing. **Jochen Saliger:** Investigation, Writing - review & editing. **Hans Karbe:** Resources, Writing - review & editing. **Gereon R. Fink:** Conceptualization, Resources, Writing - review & editing. **Jutta Stahl:** Conceptualization, Software, Data curation, Formal analysis, Writing - original draft, Visualization, Writing - review & editing, Supervision. **Peter H. Weiss:** Conceptualization, Resources, Formal analysis, Writing - original draft, Visualization, Writing - review & editing, Supervision.

## Declaration of Competing Interest

The authors declare that they have no known competing financial interests or personal relationships that could have appeared to influence the work reported in this paper.

## Footnotes

1 Please note that those 17 stroke patients included in the analysis of response-locked ERPs and the seven stroke patients excluded were not significantly different from each other in terms of demography (e.g., age) or stroke severity as revealed by non-parametric t-tests. For more information, please see the supplementary Table 1.

2 Supplementary Analysis 1 presents the main analysis of response-locked ERPs for detected and undetected errors combined, thus independent of error detection).

3 Please note that we also assessed d’ as conceptualized in signal detection theory (i.e., z(hit rate) – z(false alarms)) as this is a standardized measure for performance on the Go/Nogo task. Again, d’ was not significantly different between the LH stroke patients and healthy controls. For the sake of brevity, we do not report these results in more detail.

4 Note that we also re-analysed our data without the CSD transformation, i.e., with the raw data, in case that this might have biased the results. However, we again found the same pattern of results and no group differences on any measure (data not shown).
