# Supplementary material for: Preserved performance monitoring and error detection in left hemisphere stroke
Source: Neuroimage Clin. 2020 Jun 10;27:102307. doi: 10.1016/j.nicl.2020.102307 (PMC7306623; doi:10.1016/j.nicl.2020.102307)
Supplement: Supplementary data 1 [file mmc1.docx]

# Preserved performance monitoring and error detection in left hemisphere stroke

##### **Niessen, Ant, Bode, Saliger, Karbe, Fink, Stahl & Weiss**

**Supplemental Information**

**Supplementary Table S1**.

Differences between patient sub-groups (included vs. excluded patients) for ERP analysis

Of our 24 stroke patients, data from only 17 patients (sufficiently high number of errors) could be used to analyze the response-locked ERPs Ne/ERN and Pe, while the other seven patients (low number of errors) were excluded. In order to rule out any putative difference between those 17 stroke patients included and the seven excluded patients, we used non-parametric t-tests (Mann-Whitney-U-test for independent samples) for demographic and behavioural variables.

|  | N = 17 (high errors; included) patients  (mean ± SD) | N = 7 (low errors; excluded) patients  (mean ± SD) | p-value |
| --- | --- | --- | --- |
| age (in years) | 54 ± 12 | 63 ± 11 | p = .166 |
| Days after stroke | 63 ± 76 | 33 ± 16 | p = .710 |
| Lesion size (in pixel) | 4978 ± 4259 (n = 14) | 4559 ± 3038 | p = .999 |
| TMT-A (in ms) | 51 ± 31 | 42 ± 23 | p = .349 |
| TMT-B (in ms) | 133 ± 76 (n = 13) | 128 ± 45 (n = 6) | p = .579 |
| KAS total | 72 ± 14 (n = 16) | 72 ± 10 | p = .871 |
| ACL-k total | 29 ± 10 | 24 ± 10 | p = .166 |
| Baseline RT (in ms) | 433 ± 63 | 499 ± 58 | **p = .028** |
| ΔPES (in ms) | 95 ± 93 | 61 ± 60 (n = 6) | p = .431 |

The only significant difference between the two groups of stroke patients was observed for baseline RT with a slower baseline RT in those stroke patients, who conducted few errors, and a faster baseline RT in those patients, who conducted more errors (i.e., more than 6). As those patients, who conducted fewer errors, were generally slower, it can be assumed that they had a more cautious response tendency resulting in a different speed-accuracy trade-off.

**Supplementary Figure S1**.

Behavioral changes throughout the experimental task


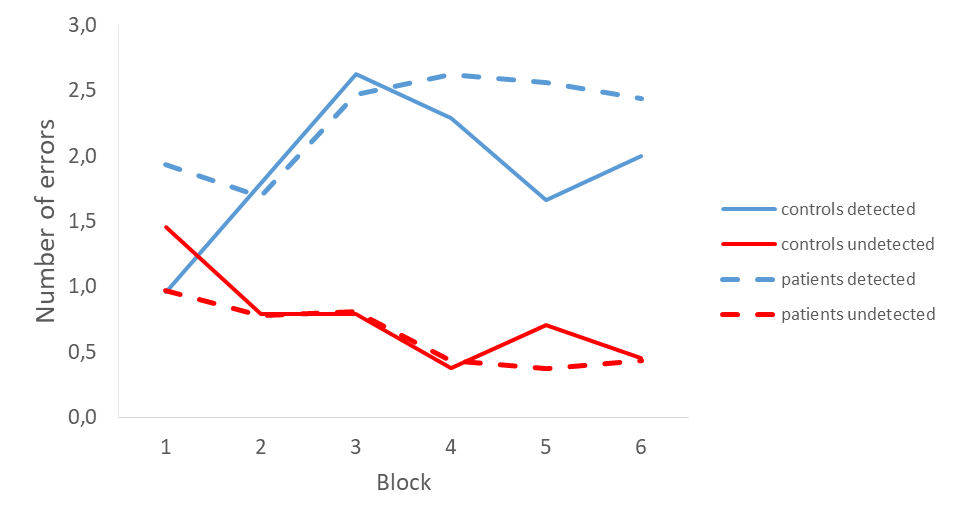


Distribution of the number of detected (blue lines) and undetected (red lines) errors across all 6 blocks are shown for stroke patients (dashed lines) and controls (bold lines) separately. A RM ANOVA revealed that there was no significant main effect of group (p = .647), nor a significant interaction with group (block x group p = .751; detection rate x group p = .369; block x detection rate x group p = .117). Interestingly, a significant interaction between block and detection rate (F(5,270) = 12.447, p < .001) suggests that the detection of errors increased with increasing time on task as indicated by an increase in detected and a decrease in undetected errors. This effect was similar for stroke patients and controls.

**Supplementary Analysis 1**

Analyses of Ne/ERN and Pe amplitudes for all errors combined (independent of error detection)

To increase statistical power, we re-analyzed the error-related ERPs for errors independent of detection, i.e., for all errors combined. Note that the pattern of results was similar to those for detected errors only: Both, the Ne/ERN and Pe, are larger for errors compared to correct responses, while there is no significant modulation by group.

RM ANOVA for Ne/ERN with group (patients and controls) and response type (correct and all errors):

- Sign. main effect response type: F(1,47) = 13.44, p < .001 (Ne/ERN is larger for errors than correct responses)
- No sign. main effect of group: F(1,47) = 1.76, p = .191
- No sign. interaction response type*group: F(1,47) = 1.08, p = .304

RM ANOVA for Pe with group (patients and controls) and response type (correct and all errors):

- Sign. main effect response type: F(1,47) = 25.11, p < .001 (Pe is larger for errors than correct responses)
- No sign. main effect of group: F(1,47) = 0.77, p = .385
- No sign. interaction response type*group: F(1,47) = 0.11, p = .916

**Supplementary Analysis 2**

Influence of apraxia and aphasia severity on task performance

Besides, we investigated whether the severity of apraxia and aphasia, as common cognitive deficits caused by LH stroke, differentially affected performance monitoring and error detection in our sample of LH stroke patients. Particularly, apraxia, which is a disorder of higher motor cognition hallmarked by “a discrepancy in accuracy between the intended action and the actual performance” (Cubelli, 2017), could affect performance monitoring. Thus, we expected to observe detrimental effects on the performance of stroke patients suffering from apraxia compared to non-apraxic stroke patients. In particular, deficits in error detection were expected, because it has recently been demonstrated that LH stroke patients with apraxia express a reduced awareness for their apraxic deficits (Canzano, Scandola, Pernigo, Aglioti, & Moro, 2014; Kusch et al., 2018), which leads to the question whether these patients also experience general deficits in monitoring their actions.

Similar to the analysis described in the main manuscript, we computed Pearson correlations and post-hoc linear regression analysis between clinical measures and task-related variables for the patient group. We used scores on the KAS and the ACL-k and correlated those with behavioral (RT, error rate, and error detection rate) and the neural measures of the Go/Nogo task (amplitude and latency of ∆N2, ∆P3, ∆Ne/ERN and ∆Pe). Eventually, we used two simple forward regressions with the following dependent and independent variables, respectively: 1. Predict RT on correct trials based on apraxia (KAS total score), 2. Predict RT on correct trials based on aphasia (ACL-k score). To correct for multiple comparisons, the adjusted critical alpha level for the correlation analysis was p = .0125 [based on four independent variables: apraxia, aphasia, and lesion size and days post-stroke (appearing in the main manuscript)].

We observed that patients with more severe apraxic and aphasic deficits had slower baseline RTs on the Go/Nogo task (*r* = -.56, *p* < .01 and *r* = -.54, *p* < .01, respectively). Note that nine patients suffered from both syndromes explaining similar correlations for apraxia and aphasia. Linear regression analyses confirmed results from the correlation analyses. Slower baseline RTs were associated with stronger apraxia (F(1,21) = 9.67, p < .005) and aphasia (F(1,21) = 8.92, p < .01), with a slightly larger proportion of explained variance for apraxia (r² = .32) than aphasia (r² = .29). Despite this interesting finding, we would like to highlight that against our expectation, the severity of apraxic deficits neither influenced error detection at the behavioral (error detection *p* = .77) nor at the electrophysiological level (for ∆Ne/ERN and ∆Pe all *p* > .13).

In summary, the presence or absence of apraxia did not modulate performance monitoring in a significant way. Apraxia, one of the most frequent cognitive deficits after LH stroke, was hypothesized to influence error detection (Klein, Ullsperger, & Danielmeier, 2013), also because LH stroke patients with apraxia previously expressed a reduced awareness for their apraxic deficits (Kusch et al., 2018; Canzano et al., 2014). The most parsimonious explanation of our current and previous findings is that the hypothesized deficits in error detection were not visible in our results, because apraxic stroke patients show deficits in awareness for *complex* actions, but their evaluation of *simple*, highly automated actions is still intact. Thus, apraxic deficits do not negatively impact the basic mechanisms of performance monitoring.

References:

Canzano, Loredana; Scandola, Michele; Pernigo, Simone; Aglioti, Salvatore Maria; Moro, Valentina (2014): Anosognosia for apraxia. Experimental evidence for defective awareness of one's own bucco-facial gestures. In: *Cortex* 61, S. 148–157. DOI: 10.1016/j.cortex.2014.05.015.

Cubelli, Roberto (2017): Definition: Apraxia. In: *Cortex* 93, S. 227. DOI: 10.1016/j.cortex.2017.03.012.

Klein, Tilmann A.; Ullsperger, Markus; Danielmeier, Claudia (2013): Error awareness and the insula: links to neurological and psychiatric diseases. In: *Frontiers in human neuroscience* 7, S. 14. DOI: 10.3389/fnhum.2013.00014.

Kusch, M.; Gillessen, S.; Saliger, Jochen; Karbe, Hans; Binder, E.; Fink, G. R. et al. (2018): Reduced awareness for apraxic deficits in left hemisphere stroke. In: *Neuropsychology* 32 (4), S. 509–515.

| **Supplementary Table S2**  Summary of EEG studies testing stroke patients.  Patient sample presents the described lesion locations of the given studies.  In the case of multiple patient groups, the number of patients in each group is stated for each group separately.  If a result is not presented, e.g., when the respective analysis was not carried out, the entry is “n.a.” (not applicable). | | | | | | | | | | | |
| --- | --- | --- | --- | --- | --- | --- | --- | --- | --- | --- | --- |
| **Author and year** | **Patient sample** | **n of patients** | **Time post stroke** | **Lesioned hemisphere** | **Task** | **Awareness test** | **Neuro-psychological assessment** | **Behavioral results (accuracy, RT, PES)** | **N2 and P3 results** | **Ne/ERN and Pe results** |  |
| Gehring & Knight, 2000 | lateral PFC | 6 | at least 1 year | 2 r, 4 l | letter discrimination task | no | no | patients worse accuracy as matched controls, patients were slower, PES not impaired | n.a. | patients reduced ΔNe/ERN |  |
| Swick & Turken, 2002 | rACC only | 1 | unknown | left | cued word-arrow Stroop test | no | no | patient worse accuracy, slower on incongruent trials, PES not impaired | n.a. | patient reduced ΔNe/ERN |  |
| Ullsperger, von Cramon & Müller, 2002 | 3 groups: lateral PFC; bilateral frontopolar (OFC); temporal lesions | 7, 6, 6 | unknown | 4 r, 9 l, 6 bilateral | speeded flanker task | no | no | PFC group similar accuracy as matched controls, PFC group slower | n.a. | PFC patients reduced ΔNe/ERN, patients smaller Pe; OFC and temporal lobe patients no abnormalities in Ne/ERN and Pe |  |
| Stemmer et al., 2004 | divers, ACA territory (e.g., medial PFC, basal ganglia, thalamus) | 5 | 168 days (mean) | bilateral | different versions of flanker task | experimenter wrote down behavioral signs of error detection | yes, e.g. d2, TMT, Tower of Hanoi (not further used) | patients similar accuracy, patients were slower | n.a. | no statistics: shape of Ne/ERN-Pe complex completely absent in 3 patients |  |
| Ullsperger & von Cramon, 2006 | 2 groups: lateral PFC; basal ganglia | 7, 9 | 5 years; 3,6 y | 2 r, 14 l | flanker task | no | no | PFC patients similar accuracy, were slower, PES not impaired; BG patients similar accuarcy , but were slower, PES not impaired | n.a. | both patient groups: reduced ΔNe/ERN, absent Pe |  |
| Turken & Swick, 2008 | ventromedial PFC | 4 | 15 y | bilateral | stroop task | no | no | patients similar accuracy, but slower RT, and PES not impaired | n.a. | patients reduced ΔNe/ERN |  |
| Wessel et al., 2014 | lateral PFC | 8 | all more than 3 years | 7 l, 1 bi | letter flanker task with subsequent novelity-oddball part | no | no | patients worse accuracy, similar RTs, PES not impaired | P3a for novelty, response-locked | patients reduced ΔNe/ERN, in patients no Pe detectable |  |
| Maier et al., 2015 | 2 groups: rACC and ventromedial PFC; brain damage control (BDC) group with lesions outside ACC | 7,7 | at least 1 y | 4 r, 4 l, 6 bi | flanker task | yes | yes, MMST and standardized test battery (not further used, note that patients performed in a normal range) | patients similar accuracy to BDC but better than controls; slower than BDC and controls, PES not impaired - PEA impaired, similar error detection | P3 similar for patients and controls | rACC patients reduced ΔNe/ERN compared to BDC and controls; rACC no abnormalities in Pe |  |
| Niessen et al. (current study) | MCA territory | 24 (17) | 54 days (mean) | left | Go/Nogo task | yes | yes (MMST, clock test, BDI, handedness, KAS, ACL-k, mRS, TMT) | patients similar accuracy, similar RTs, PES not impaired - similar error detection | N2 later in patients, P3 smaller | patients similar ΔNe/ERN and Pe compared to controls |  |
